# Could fNIRS Promote Neuroscience Approach in Clinical Psychology?

**DOI:** 10.3389/fpsyg.2016.00456

**Published:** 2016-03-30

**Authors:** Roberta Adorni, Alessia Gatti, Agostino Brugnera, Kaoru Sakatani, Angelo Compare

**Affiliations:** ^1^Department of Human and Social Sciences, University of BergamoBergamo, Italy; ^2^Department of Engineering and Applied Science, University of BergamoBergamo, Italy; ^3^Human Factors and Technology in Healthcare, University of BergamoBergamo, Italy; ^4^Department of Electrical and Electronic Engineering, College of Engineering, NEWCAT Research Institute, Nihon UniversityTokyo, Japan

**Keywords:** fNIRS, prefrontal cortex, verbal fluency task (VFT), major depressive disorder, relapse

## Introduction

The purpose of the present commentary is to consider new perspectives of investigation methods used in clinical psychology studies. We propose that functional Near-Infrared Spectroscopy (fNIRS) has several important advantages over currently used methods. This technique opens the opportunity to transfer psychophysiological evidences in clinical settings, for example to evaluate psychophysiological underpinnings of depressive symptomatology. Indeed, fNIRS technology might be promisingly used to find out objective biomarkers of relapse risk of depression after pharmacological and psychotherapeutic treatments.

## fNIRS is a valuable tool for the investigation of psychological processes in ecological contexts

The challenge of future research in clinical psychology is to describe psychophysiological underpinnings of psychopathology. With this ambitious purpose in mind, we think that, on one hand, clinical psychologists should start to take into consideration the relevance of the psychophysiological components of psychopathology. On the other hand, neuroscience research should start to take into consideration the importance to provide instruments suitable to investigate Central and Autonomous Nervous System in more ecological and clinically usable contexts.

Neuroscience research has provided evidence of the cerebral regions involved in psychological processes. Neuroimaging techniques like fMRI or PET revealed precious information, as they provide accurate and valid evidence about the cerebral regions involved in specific cognitive and affective processes. Among cortical structures, the Prefrontal Cortex (PFC) seems to be the most involved in the identification and in the production of affective responses (Phillips et al., 2003), as well as in emotional regulation and executive functioning (Ochsner and Gross, 2005; Hofmann et al., 2012; Kolb et al., 2012; Okon-Singer et al., 2015). Despite being very effective, these techniques have many technical constraints, economic costs and expertise requirements that make them unsuitable for a widespread use in clinical psychology. Different instruments are necessary to overcome all these issues.

fNIRS is a technique that allows measurement of brain activity changes by means of probing the concentration and oxygenation of cerebral hemoglobin. Recent studies have demonstrated that fNIRS is useful to evaluate the association between the PFC activation and executive functions (for a review see Moriguchi and Hiraki, 2013) as well as emotion processing (Doi et al., 2013). Compared with other neuroimaging techniques, like fMRI and PET, or electrophysiological techniques, like ERPs, fNIRS has respectively poor spatial and temporal resolution, and depth resolution is limited to cortical layers of the brain (for a detailed discussion on the limits of fNIRS, see Dieler et al., 2012), but it offers several advantages. First, fNIRS is less sensitive to external noise sources, like subject movements. As a result, during fNIRS recording, individuals can speak and interact with others like a common everyday social situation. Otherwise, fNIRS it is not strictly dependent on the environmental electromagnetic field, resolving some limits of both fMRI and ERPs. Secondly, fNIRS requires less time, and less physical and psychological burden for the preparation of the subjects and the application of the probes. In this way, brain activity changes can be measured out of the laboratory, in more ecological and clinically usable contexts. Finally, it is less onerous if compared to the other neuroimaging techniques, both in terms of instruments costs and in terms of the expertise required to record and analyze the signal.

In summary, fNIRS technique is easy to use and transportable, is noise-resistant and minimally invasive and can be successfully employed in various neurophysiological areas. For example, due to its advantages it has been recently used to investigate PFC activity both during mental stress responses and resting state (Tanida et al., 2004, 2007, 2008; Sakatani, 2012; Ishikawa et al., 2014). Thus, we are confident that fNIRS might represent a resource for more ecological studies, which could be difficult to realize with more sophisticated but restrictive instruments. Another important consideration is that some aspects of psychiatric disease, in particular risk of relapse, are difficult to evaluate using traditional methods like interviews and self-reported measurements: therefore, in this commentary we suggest the use of fNIRS technology to identify objective biomarkers of a relapse risk of psychopathology. Considering the abundance of fNIRS studies on depression, we focused on this psychiatric disorder.

## The use of fNIRS in psychiatric research: state of art

fNIRS technology has been used in the last 15 years to assess hemodynamic responses of various psychiatric conditions, with Major Depressive Disorder (MDD) being one of the most studied. According to the Diagnostic and Statistical Manual (American Psychiatric Association, 2000) MDD is characterized by pervasive and persistent low mood, anhedonia, as well as by a constellation of cognitive symptoms and impairments. Abnormal emotion processing is a core feature of MDD. Nevertheless, fNIRS technology is not suitable to explore the activation of deep cerebral structures that are known to be closely involved in emotion processing (Rive et al., 2013), but just cortical brain structures, like the PFC. For this reason, studies performed with fNIRS technology have focused on the cognitive characteristics of MDD. These characteristics seem to play a primary role in the development, susceptibility and the maintenance of depression (Levin et al., 2007; Clark et al., 2009), and they are likely to persist even after the depressive symptoms remission (Levin et al., 2007; Tomioka et al., 2015). Past review and meta-analyses focused on neuroimaging studies, in particular fMRI or PET, showed a straight association between MDD and both functional and structural abnormalities in various PFC areas (for a review see Rive et al., 2013; Zhang et al., 2013). The dorsolateral prefrontal cortex (DLPFC) seems the region most involved in the cognitive symptoms of MDD, with a notable hypoactivation in depressed patients during specific cognitive tasks (Snyder, 2013). Indeed, DLPFC activation has been associated to executive functions, term used to describe higher-level cognitive processes, such as working memory, attention, and problem solving, which supervise other cognitive domains (Snyder, 2013). Tasks that typically assess one or more executive functions (i.e., verbal fluency or working memory tasks) evidenced a constant hypoactivation of this area in depressed patients, when compared to healthy controls (for a recent meta-analysis see Zhang et al., 2015).

Consistent with neuroimaging studies, the majority of the fNIRS studies explored the PFC activation during verbal fluency tasks on different subgroups of patients with depressive symptoms (see for example, Suto et al., 2004; Pu et al., 2008; Liu et al., 2014; Tomioka et al., 2015). These studies obtained similar results, evidencing that these patients showed reduced activation in the PFC during a verbal fluency task as compared to healthy controls. Hypoactivation of the PFC during cognitive tasks is so strictly interconnected with depression that the frontal hemodynamic activation patterns associated to verbal fluency tasks have been recently used to discriminate between patients affected by depressive symptoms and healthy subjects. Indeed, Takizawa et al. (2014) found that the PFC activity of patients affected by depressive symptoms, when compared with that of healthy controls, was characterized by a significant hypoactivation. The amount of this hypoactivation was more or less accentuated based on the general psychiatric condition of the patients. Importantly, the PFC hypoactivation in MDD patients in response to a verbal fluency task seems to persist even after the remission of symptomatology following antidepressant therapy (Tomioka et al., 2015), leading to the hypothesis that such hypoactive response could be used as an objective biomarker of relapse risk of depression after pharmacological and psychotherapeutic treatments. More studies are necessary to confirm this hypothesis, considering that different findings have been described. For example, Ohtani et al. (2015) found that the PFC hypoactivation in MDD patients decreased over time, as symptomatology remitted (assessed as improvement in social adaptation).

## Future directions: could fNIRS detect vulnerability factors of depressive relapse?

During the past few years, clinicians focused on MDD treatment have paid more attention to risk of relapse and recurrence prevention. Whereas at least 45% patients affected by MDD experienced recurrences (Kruijshaar et al., 2005), the importance of this aspect in depression's treatment is no longer questioned. According to the National Institution for Health and Clinical Excellence (NICE), psychotherapy as Cognitive Behavioral Therapy (CBT) or Mindfulness-Based Cognitive Therapy (MCT), as well as antidepressant treatments, need to be provided for long time after remission (NICE Clinical Guidelines, 2009). Indeed, a recent review confirmed the effectiveness of psychological intervention (both CBT and MCT, as well as Interpersonal therapy) in preventing the reemergence of symptoms (relapse) or the onset of a new episode following a period of recovery (recurrence, Biesheuvel-Leliefeld et al., 2015). These data are important but concerning in terms of cost-effectiveness for the health care system. The understanding of what are the predisposing factors of relapse may allow to personalize risk assessment following remission and to design intervention addressed to known mediators of relapse. Considering that relapse and recurrence seem to share part of the vulnerability factors, and that the two terms are often confused (Farb et al., 2015), in the present commentary we focus only on relapse.

Literature findings on which factors contribute to increase risk of relapse is not exhaustive yet. Nowadays, personality, genetics and clinical history of MDD have been established as vulnerability factors (Farb et al., 2015). Importantly, some researchers in clinical psychology underlined the relevance of cognitive vulnerability factors, such as dysfunctional beliefs, as significant predictors of relapse. For example, Segal et al. (2006) assessed dysfunctional beliefs associated to sad mood induction (obtained combining “sad music” and recall of sad autobiographical episodes), and they found that this mood-linked cognitive reactivity seems to be a significant predictor of relapse. Therefore, as already mentioned, the investigation of depression should not be uniquely focused on emotion and affect, but also on dysfunctional thoughts, which are based on specific cognitive processes. In our opinion, future researchers should focus on the possible links between clinical psychology and cognitive neuroscience literature, proposing integrated models that take into consideration different conceptual levels, namely neuronal circuits, cognitive processes, and higher level thoughts. We are confident that fNIRS could be a suitable technique to explore cognitive processes that might represent vulnerability factors for relapse occurrence. So far, fNIRS literature approached to the study of depression analyzing “non-specific” cognitive processes (i.e., using verbal fluency task to assess executive functions). A more analytic approach—grounded on the characteristics of depressive symptoms—should be considered, in order to understand which specific cognitive processes might represent vulnerability factors of relapse. A significant example of such an articulated approach is the one proposed by Farb et al. (2015). The authors described a two factor model of relapse/recurrence vulnerability in Unipolar Depression that emphasizes especially neuronal aspects. This model analyzes the well-known cognitive process of “rumination” and consider a separate role of two specific vulnerability factors in the occurrence of depression sensitization, namely dysphoric attention and dysphoric elaboration. These concepts suggest that depressed patient tends to fix to negative features of events (dysphoric attention) and to create strong associations between negative events and a centralized self-concept, leading to generalized dysphoric attitudes toward the self, the future and the world (dysphoric elaboration). Among the results, they evidenced that dysphoric attention was associated to hypoactivation of the DLPFC, and that dysphoric elaboration was associated to hyperactivation of the medial PFC. Therefore, the focusing on those cognitive processes that are really involved in depressive symptomatology could represent the methodological and perspective change necessary to find valid, reliable and clinically useful biomarkers of relapse risk.

In conclusion, new perspectives of investigation methods used in clinical psychology studies have been discussed. fNIRS could be a precious tool to investigate brain activity in more ecological and clinically usable contexts. Considering its technical advantages, it might represent a resource for clinical studies, which could be difficult to realize with more sophisticated but restrictive instruments. Taken together, the studies mentioned above suggest that, in the future, fNIRS might be used to identify psychophysiological underpinnings of psychiatric symptoms. We are confident that, following a more analytic and clinically oriented approach (as the one proposed by Farb and colleagues), fNIRS could be a suitable technique to explore cognitive vulnerability factors possibly involved in relapse occurrence that are currently difficult to evaluate, and consequently personalize care in clinical setting.

## Author contributions

AC conceived the topic. RA, AG, and AB developed the topic and wrote the manuscript. KS supervised the manuscript. All authors read and approved the final manuscript.

## Funding

Paper was funded by “Bergamo for Health in Aging and Globalized Society” project funded by University of Bergamo within the call for research ITALY® — Italian TALented Young Researchers, Action 2 (2012-2013).

### Conflict of interest statement

The authors declare that the research was conducted in the absence of any commercial or financial relationships that could be construed as a potential conflict of interest.
